# 

**Published:** 1872-05

**Authors:** 


### CONTENTS.

### COMMUNICATIONS.

Poisoned by Mercury from a Tooth Filling .......... 189
Teeth Fibrous .......... 194
Interdental Splint—With Case in Practice .......... 198
Reasonable Talk on the Subject of Filling Teeth .......... 200
Unnecessary and Unkind .......... 206
Nitrous Oxide .......... 208
The Vehicle for Chloral .......... 209
Correspondence .......... 210

### PROCEEDINGS OF SOCIETIES.

Minutes of the 28th Annual Meeting of the Mississippi Valley Dental Society .......... 212
Mississippi Valley Dental Association .......... 217
Second District Dental Society of New York .......... 219

### SELECTIONS.

The Treatment of Inflammation .......... 221

### EDITORIAL.

Inflammation .......... 228

The Rubber Patent Case .......... 230

What Will be the Result .......... 230

### OBITUARY.

The Departed .......... 231
Dr. Hurd .......... 230
R. W. Varney .......... 231
Tribute of Respect .......... 232

